# Voices from the emergency department: A theoretical framework analysis on patient experiences of care in emergency departments of Newfoundland and Labrador, Canada

**DOI:** 10.1371/journal.pone.0342555

**Published:** 2026-02-09

**Authors:** Aswathy Geetha Manukumar, Nahid Rahimipour Anaraki, Holly Etchegary, Anna Walsh, Oliver Hurley, Christopher Patey, Paul Norman, Shabnam Asghari

**Affiliations:** 1 Centre for Rural Health Studies, Faculty of Medicine, Memorial University of Newfoundland, St. John’s, Canada; 2 NLSUPPORT, Memorial University of Newfoundland, St. John’s, Canada; 3 Faculty of Medicine, Memorial University of Newfoundland, St. John’s, Canada; 4 Discipline of Family Medicine, Faculty of Medicine, Memorial University of Newfoundland, St. John’s, Canada; 5 Newfoundland and Labrador Health Services, Carbonear Institute for Rural Reach and Innovation by the Sea, Carbonear General Hospital, Carbonear, Canada; University of Saskatchewan, CANADA

## Abstract

**Background:**

Patient experience in the emergency department (ED) encompasses different aspects of care, such as respect, communication, timeliness, shared decision-making, and care transition. The inherently stressful ED environment presents additional challenges for providers in ensuring a positive patient experience. Studying patient experience allows health systems to recognize areas of care that need improvement and introduce strategies to improve patient-centred care.

**Objectives:**

To explore the patient experience of emergency department care in Newfoundland and Labrador (NL), Canada.

**Methods:**

This qualitative study collected data from patients who visited two urban and two rural EDs in NL via telephone surveys and semi-structured interviews. Five researchers used a theoretical framework, symbolic interactionism, to analyze open-ended survey responses and semi-structured interviews. Patient research partners were consulted to ensure the themes reflected their lived experiences.

**Results:**

A total of 836 responses were analyzed (831 survey responses, five semi-structured interviews), leading to six key themes on patient experience. They are: (1) Mutual respect and trust in providers, (2) Timeliness of care, (3) Communication, (4) Comfort and accommodations, (5) Information sharing and decision-making, and (6) Continuity of care.

**Conclusion:**

Our findings show an immediate need to improve the patient experience of ED care and highlight areas within these themes that could be targeted for improvement. We recommend regular training programs for healthcare providers to improve their interpersonal skills, the addition of patient navigators, and modifications to the triage system for vulnerable populations to improve patient experience and provide patient-centred care.

## Introduction

Patient experience is defined as “the sum of all interactions (of patients) shaped by the organizational culture that influences patients’ perceptions across the continuum of care” (Wolf et al., 2021) [1]. In other words, it is not limited to individual clinical interaction at a single care setting, but encompasses every encounter a patient has with the healthcare organization, including structural conditions [1]. As such, patient experience spans all aspects of care, reflecting how patients perceive, interpret, and evaluate their care during a hospital visit and has become crucial area of research for health leaders [1,2]. According to the Canadian Institute of Health Information, it is essential to evaluate patient experiences as it allows health systems to understand their performance, introduce quality measures, and better meet patients’ needs and expectations [3].

A chaotic emergency department (ED) creates additional challenges in providing a positive patient experience [4,5]. EDs are fast-paced, unpredictable, frequently crowded and noisy [4,5]. According to the National Ambulatory Care Reporting System, over 16.1 million unscheduled ED visits were reported in Canada between April 2024 and March 2025 [6]. Patients typically arrive at the EDs requiring urgent care, undergo multiple unexpected assessments and treatments, some of which may be uncomfortable or intrusive, and there is often not enough time to build a rapport with providers [4,5].

A 2018 systematic review on ED patient experience showed that one of the most important aspects of patient experience is staff-patient communication, followed by wait times, empathy and compassion of ED staff, demographic characteristics of patients, and medical competence of the staff [7]. These themes have been consistently highlighted in other studies [4].

Interpersonal and informal communication are key in defining the patient experience. Patients appreciate it when providers pay full attention to them, acknowledge their concerns, and calmly provide clear responses [4,7–9]. Lengthy wait times often result in frustration and dissatisfaction among patients, and they have expressed a preference for receiving timely updates about delays and having a more comfortable ED environment to help alleviate the stress of waiting [4,7–9]. Patients also expect empathy and compassion from providers [4,7,9]. When patients are supported in coping with uncertainty during their ED visits (e.g., through pain relief), and involved in decisions about their care, it leads to a more positive care experience [4,7,9]. These themes are also reflected in the literature on patient-centred care (PCC), reinforcing their significance as fundamental components of high-quality care [9–11].

Age also influences patients’ care experiences, as older adults often express concerns about losing their independence due to chronic conditions or feeling lonely, abandoned, and depersonalized in the ED [7]. However, several studies have shown that older patients appeared less likely to voice dissatisfaction overall and were particularly understanding of ED conditions [7]. Furthermore, there is limited evidence on the impact of gender on patients’ ED care experiences [7,12]. Finally, patients were often attentive to clinical processes, and when they perceived their providers as operating skillfully and efficiently, it positively impacted their overall care experience [7].

Despite the importance of studying patient experiences, our literature search showed a lack of research on care experiences in Canadian EDs. The province of Newfoundland and Labrador (NL) is predominantly rural [13–15], with a significant proportion of its population aged 65 or older, 4% higher than the national average (2021 Census) [16]. The NL health system serves widely dispersed rural communities, with challenges in timely access to medical care driven by system-level issues such as physician shortages and long-standing underfunding [13,17]. To our knowledge, our study is the first to explore ED patient experience in NL.

## Materials and methods

### Qualitative approach and context

This qualitative study used thematic analysis to explore patients’ care experience visiting four EDs (two rural, two urban) within the Eastern Health region of NL [15]. The two rural EDs each serve populations of approximately 32,000 and 20,400 people, with an average of 21,600 and 20,400 visits per year, respectively [18,19]. In contrast, the urban EDs serve the broader population of Newfoundland and Labrador, averaging 54,000 and 38,400 visits annually [18,19]. Staffing differs between settings, with rural EDs supported by 6–10 physicians and 12–15 nurses, while the urban sites have 40 physicians and 55–70 nurses [18,19].

We used a convergent design [20] and integrated data collection through telephone surveys and semi-structured interviews, allowing a more comprehensive analysis of patient experience. The telephone survey is available as S1 File, and the semi-structured interview guide is available in S2 File.

### Ethics

This study is part of a “SurgeCon” project implemented at four EDs in NL [21,22]. SurgeCon is an extensive research endeavour to improve ED flow, patient experience, and patient satisfaction in NL. Further project details are available via Anaraki et al. [21] and Mariathas et al. [22] (Trial registration number: NCT04789902). NL’s Health Research Ethics Authority (HREA) provided ethics approval for this research (HREB #2019.264).

### Patient involvement

Interpretation of results and final manuscript preparation were completed in consultation with all team members, including the patient research partners (PRPs). PRPs are patients, caretakers, or guardians with knowledge and experience of the NL EDs [18,21–23]. They were recruited through Newfoundland and Labrador Support for People and Patient-Oriented Research and Trials (NL SUPPORT), personal networks of individuals with ED experience, and from among survey participants who expressed interest in contributing to the research [18,21–23]. Since SurgeCon’s inception, the PRPs have engaged with the team on different parts of the project, including guiding the development of data collection techniques and creating knowledge translation and exchange materials [18,21–23].

### Sampling and data collection

Data were collected through open-ended questions in a telephone survey and through follow-up qualitative, semi-structured interviews [18,23]. The telephone surveys were conducted from March 1, 2021, to July 27, 2023, and the semi-structured interviews were conducted from May 1, 2024, to July 29, 2024 [18,23]. Male and female research assistants (RAs) with undergraduate degrees collected data after completing ethics training and SurgeCon’s interview training [18,23].

Participants in the survey were randomly selected from patients who visited the four EDs during the data collection period [18,21–23]. Eligibility criteria included any patient who visited these EDs during the specified period, with no additional inclusion or exclusion criteria [18,21–23]. Randomization was based on the time and date of the patient’s visit [18,21–23]. One of the RAs contacted the selected patients within 48 hours of their ED visit. The RAs contacted at least 25 patients per ED each month and attempted up to three calls per patient before moving on to the next. In total, at least 1,575 surveys were offered during the study period. Additional details on participant selection are available in our prior publications [18,21–23]. Once contacted, the RAs provided an overview of the study, and obtained informed verbal consent if they were interested in participating [18,23]. In cases where minors or adults were unable to provide consent, their parents/guardians/caretakers consented to their participation [18,23]. The consent was recorded and saved in a secure patient log form, and prior to proceeding with the survey, the interviewer was required to confirm, by checking a designated box, that the consent form had been read and explained to the participant, and that the participant demonstrated awareness and understanding of the research study [18,23]. This process was approved by the HREA for receiving and recording informed consent for this study.

The survey was developed based on existing patient experience surveys [18,21–23], such as the Canadian Patient Experience Survey – Inpatient Care (CPES-IC) [3]. It was co-developed with PRPs and pilot-tested with ten volunteers [18,21–23]. Open-ended survey questions allowed patients to share additional insights regarding various aspects of their care [18,23]. This included suggestions for improvements, concerns about their experience, recommendations for improving the ED environment, details on visit-related costs, and reflections on particularly positive or negative aspects of their ED visit [18,23]. Finally, at the end of the survey, the RA invited patients to participate in a follow-up in-depth semi-structured interview [18,23]. All participants who completed the telephone survey were entered into a random prize draw, and 2% of the participants had the chance to win gifts valued between $200 and $500 [18,23].

The semi-structured interviews of interested participants were conducted later by phone at the participants’ convenience [18,23]. Similar to the survey, the interviews started by providing participants with an overview of the project and obtaining informed consent [18,23]. The interview guide was co-created with PRPs based on the survey responses and patients’ lived experiences and perspectives [18,23]. These interviews let us capture more nuanced insights into patients’ experiences. Interview questions were centered mainly on respect, trust, communication, information sharing, and the comfort of the environment. These interviews lasted up to 60 minutes and were recorded and transcribed verbatim [18,23]. All the participants were compensated with a $25 gift card [18,23].

This approach enabled a thorough identification and analysis of themes, providing a comprehensive understanding of key issues affecting patient experiences in EDs. Researchers could not access identifiable patient information, and data was stored and analyzed in Microsoft Teams [18,23].

### Analysis

In this study, thematic analysis with a convergent design [20], was employed to examine open-ended questions from the survey and semi-structured interviews to extract themes related to patient experiences in the ED.

### Theoretical framework

Symbolic interactionism (SI) is a theoretical perspective that explores how social reality is constructed and sustained through the ongoing interactions and shared meanings between individuals [24]. SI allows us to explore lived experiences and how people assign meaning to themselves and others while engaging with their social environment [24]. In the context of health research, SI emphasizes how individuals interpret and assign meaning to health and illness, how these meanings are formed and negotiated through social interactions, how they contribute to shaping personal identity and self-concept, and how they ultimately guide behaviour and decision-making [24]. Since patient experience encompasses the full range of interactions encountered across the continuum of care, applying SI enables a deeper exploration of how patients construct meaning from these encounters and how such meanings influence and shape their overall healthcare experience [1,24].

### Rigor

We followed the six steps suggested by Braun and Clarke (2006) [25] for conducting the analysis. The first step involved analyzing the responses to the open-ended survey questions [18,23]. Five researchers (AM, NA, AW, OH, SA), with experience in qualitative health research, independently coded the data to ensure the study’s credibility [18,23]. The team then compared their analyses and generated the initial themes [18,23]. Weekly discussions and clarification resolved disagreements around the codes and themes [18,23]. This also allowed for refining and defining the themes, improving consistency, and cohesiveness.

An RA conducted the semi-structured interviews [18,23]. The same group of co-authors analyzed those interviews, using the identical analytic approach [18,23]. Themes derived from these responses were compared with those identified from the open-ended survey responses to support triangulation and cross-validation of the findings [18,23].

Finally, peer validation was conducted through an iterative review process [18,23]. The group of co-authors responsible for coding and data extraction presented preliminary codes and themes to a multidisciplinary team, including those with expertise in patient-centred care (HE), healthcare professionals (CP, PN) and PRPs [18,23]. Feedback from these groups was integrated into the analysis before finalizing the themes [18,23]. We concluded the semi-structured interviews once thematic saturation was achieved, as indicated by repeated patterns in the data and determined through team consensus [18,23]. Finally, all the findings were presented to PRPs during analysis and manuscript preparation to verify the accurate representation of their experiences [18,23].

## Results

### Patient characteristics

A total of 831 patients completed the survey during the study period. Most participants were females (62%), 61 years or older (41%); Table 1. Five of these patients also participated in the semi-structured interviews, including four females (80%) and one male (20%). Overall, we analyzed 836 responses.

**Table 1:** Demographic characteristics of survey participants and responses collected (*N* = 831).

Characteristic	Overall *N*, %
Gender	
Female	518 (62)
Male	313 (38)
Age in years	
0–20	61 (7)
21–40	153 (18)
41–60	274 (33)
≥ 61	343 (41)
ED location	
Rural	399 (48)
Urban	432 (52)
The final two open-ended survey questions	
What would you change in the care you received, if at all?	817 (98)^a^
Is there anything else you would like to tell us about your experience at the ED?	679 (82)^b^

^a^14 participants did not answer the question

^b^152 participants did not answer the question

Our study achieved maximum variability in age distribution and a balanced representation of patients from rural and urban EDs. The analytic process, guided by SI, centered on participants’ lived experiences and subjective interpretations of care. As the analysis progressed from open-ended survey responses to semi-structured interviews, no new themes emerged. Instead, existing themes were consistently reinforced across participants, reflecting coherence in how care experiences were perceived. The repetition of key ideas across multiple data sources and participant groups indicated that thematic saturation had been reached.

### Themes on patient experience of ED care

Using a thematic analysis approach grounded in SI, six key themes most relevant to patient experience research were identified from the semi-structured interviews and open-ended survey responses: (1) Mutual respect and trust in providers, (2) Timeliness of care, (3) Communication, (4) Comfort and accommodations, (5) Information sharing and decision making, and (6) Continuity of care. These themes reflect how patients constructed meaning around their ED experiences.

1. Mutual respect and trust in providers

Patients highly valued respect from providers, which fostered mutual respect and enhanced trust in their expertise and clinical judgment. When providers demonstrated respect and engaged with patients in a considerate manner, patients reported a positive experience.

*“[Patient] was very happy with the care that he received. He said that he hadn’t been to an ED before in his life and was surprised with the excellent care he received. He said that he was treated with great respect and that the ED staff handled his health concerns very well.”* – Survey response from a male, ≥61years old, who visited an urban ED

Conversely, when providers failed to acknowledge patients or disregarded their concerns, it resulted in a lack of respect and increased mistrust in the healthcare system.

*“[Patient] was very unhappy with the care she has received at the ED… she felt like her health concern was an emergency that was not taken seriously… [patient] spoke often about being worried about dying if she were to go in with something more serious and had little trust in the medical system.”* – Survey response from a female, 41–60 years old, who visited an urban ED

*“[Patient’s mother] said…she felt dismissed by the doctor since it was the second time bringing her son in for the same issue…The doctor made her feel like she was wasting resources and the time of the ED staff, even though 811 told her to go back to the ED.”* – Survey response from the mother of a male patient, 0–20 years old, who visited a rural ED

*“[Patient] was not treated with any respect or empathy…refused to treat her for her pain after it didn’t subside during the first treatment…the doctor called [patient] an addict in front of the ED…was embarrassing…they didn’t take her pain seriously…ED experiences have given her major anxiety over the years…and they [providers] don’t treat her with empathy for her mental health problems.”* – Survey response from a female, 21–40 years old, who visited a rural ED

Our data also suggests that female patients (≥21 years old) or mothers raise more concerns about mutual respect and trust in providers.

2. Timeliness of care

Timely access to care was one of the critical components of the patient experience of ED care. Although some patients surprisingly had a quick visit with minimal waiting, this was not the typical experience for most patients. Patients expressed surprise at how much quicker their visit was compared to previous visits, indicating that their expectations for the care experience were exceeded.

*“[Patient] said her experience at the ED was great…she got better care than she even expected to get and that the staff was all quick and thorough with her care…the ED staff there was the best she has ever had…”* – Survey response from a female patient, 41-60 years old, who visited an urban ED

*“[Patient’s mother] stated she was quite satisfied with the wait time as she had expected to wait for much longer…”* – Survey response from the mother of a male patient, 0–20 years old, who visited a rural ED

However, many patients discussed having to wait several hours for emergency issues and having to leave the ED due to excessive wait times.

*“[Patient] mentioned she waited for about 8 hours, constantly vomiting the entire time, and still there was no update on wait time given to her, neither did any nurse check on her. She eventually gave up on waiting and called an ambulance to go to (a different ED) where she was able to receive treatment.” –* Survey response from a female, 21–40 years old, who visited an urban ED

Furthermore, patients often had to wait hungry and thirsty as the staff did not inform them of the wait time or check on their well-being.

*“I want[ed] to go. I didn’t really feel comfortable because she [the staff] couldn’t promise me that I wouldn’t miss the call to bring me in, so I basically had to wait and be hungry…”* – Semi-structured interview response from a female, 41–60 years old, who visited an urban ED

This led many patients to lose trust in the healthcare system, causing them to avoid returning to the ED unless their condition was critical—sometimes even then, they hesitated.

*“[Patient] was in the ED for hours…with severe pain that was not taken seriously… had to return to the ED later that night after his pain had gotten worse…waited overnight in the ED without being seen…has lost all respect for the NL health system…will “never, ever, ever” use the NL health system again for serious health concerns.”* – Survey response from a male, ≥61 years old, who visited an urban ED

Patients also reported wanting the staff to check on them more often while waiting and to be more attentive to their worsening conditions.

*“No staff checked on the comfort of anybody in the waiting room for the entire time spent there (>4 hours…The staff was very rude, and the triage nurse was extremely cold and irritated…A man passed out, and nobody rushed to him. It seemed like they thought he was doing it on purpose to get faster service…”* – Survey response from a female, 41–60 years old, who visited an urban ED

Finally, patients identified overcrowding due to a lack of resources as a reason for lengthy wait times.

"*[Patient's mother] said that there needs to be better use of non-urgent care clinics and healthcare services outside of the ED to free up space and resources in the ED...*" - Survey response from the mother of a female, 0 - 20 years old, who visited a rural ED

3. Communication

Communication encompassed adequate contact between providers, hospital staff, patients, family members, and support persons. Patients appreciated providers listening to them attentively and taking their concerns seriously.

“*[Doctor] was amazing, listened attentively, and took issues seriously that other doctors had brushed off as unimportant.”* – Survey response from a female patient, ≥61 years old, who visited a rural ED

However, several patients were concerned about their providers’ lack of consistent communication, feeling rushed, and being dismissed.

*“[Patient] said that the staff seemed very rushed, and it was as if they didn’t want him to ask any more questions about the care.”* – Survey response from a male, 21–40 years old, who visited an urban ED

Another major concern was patients’ health literacy. Difficulty understanding medical terms indicated that some providers were not communicating effectively.

*“[Patient] had to ask the doctor to explain the diagnosis in layman’s terms since the doctor was using too much medical jargon when speaking with her.”* – Survey response from a female, 0–20 years old, who visited a rural ED

Finally, several instances of miscommunication led to delayed care or testing, putting patients at greater risk of severe illness or adverse consequences.

*“[Patient] waited overnight in the ED without being seen or checked on by anyone... another doctor prescribed him medication for a condition that he did not have. After the tests came back, it showed that he had a potentially fatal and very urgent health emergency that had been ignored and missed in the previous day’s bloodwork review.”* – Survey response from a male, ≥61 years old, who visited an urban ED

4. Comfort and accommodations

Patients valued quiet waiting areas that provided ample space and comfortable seating, ensuring a more relaxed and accommodating environment.

“*Oh, it [the waiting room]is really, really nice. The chairs are really nice, and it’s just a very quiet atmosphere there…like the people waiting…pretty quiet, don’t have a whole lot to say…”* – Semi-structured interview response from a male patient, 41–60 years old, who visited a rural ED

However, most patients felt their comfort and accommodations went unrecognized or were disregarded by the providers.

*“[Patient] stated chairs in the waiting room were very uncomfortable and aggravated his disability, which “nearly killed” him…went to the reception a few times, asking if there was somewhere more comfortable for him to sit…was dismissed.”* – Survey response from a male, 41–60 years old, who visited a rural ED

The discomfort issues extended beyond individual concerns, with many patients expressing concerns for others in the ED due to inaccessibility to water, food, easy wayfinding, and internet.

*“[Patient] stated there is no access to anything in the waiting room. Things like water, snacks, etc... it was not really that much of an issue for her, but for someone who is diabetic and has no one with them to assist, it might be concerning.”* – Survey response from a female patient, 41–60 years old, who visited a rural ED

The lack of space in the waiting areas and curtained cubicles also caused immense privacy concerns. Many patients were reluctant to share personal information, while bystanders often felt uncomfortable overhearing or witnessing others’ medical issues and conversations with healthcare providers.

*“[Patient’s mother] said the care [patient] received was careless…they did not do what they should have done to care for her…the NP she had was not very helpful…the room she was in had the curtains pulled back and there were other patients around so she didn’t have any privacy which was uncomfortable…overall it was a very negative experience…”* – Survey response from the mother of a female patient, 0–20 years old, who visited an urban ED

Additionally, many wished there were a separate space or additional support for individuals who could not withstand the waiting room environment or were disruptive to others. This included patients with mental health issues, substance issues, or behavioral problems.

*“[Patient] has sensory issues related to autism and ADHD; because of this, the waiting room was very overwhelming for her. There were few to no accommodations in place for her. She was in an overstimulating environment for over 4 hours. Upon asking for help, she was allowed to be put in a quiet room (“the COVID room”), which did not ease her overstimulation...* – Survey response from a female patient, 41–60 years old, who visited an urban ED

*“While [patient] was in the waiting room, there was another patient there who she described as ‘irate’ and very disruptive of the environment. She was quite concerned and uncomfortable because he was yelling at the staff and being incredibly disrespectful. She was upset about the incident and suggested having a patient in a state like that treated quicker or kept in a separate area while waiting for care so the other patients wouldn’t be disrupted.”* – Survey response from a female patient, ≥61 years old, who visited an urban ED

5. Information sharing and shared decision-making

Patients needed detailed information about their treatment plans and to be included in decisions about their care.

*“[Patient’s] experience at the ED was great…The ED staff there was the best she has ever had compared to other ED experiences she has had at different hospitals…they gave her great information and direction about the care she received...”* – Survey response from a female patient, 41–60 years old, who visited an urban ED

In contrast, many patients shared a need to receive more information about treatment and follow-up.

*“The staff should give patients more information regarding their health when they visit the EDs rather than giving them medications and sending them home without much plan for follow-up or treatment plan.”* – Survey response from a male patient, 21–40 years old, who visited a rural ED

Furthermore, many patients felt their voices were not heard in decisions about their care, with their needs and feedback often overlooked, leading to additional challenges.

*“The doctor wasn’t thorough enough…did not do enough testing…had to advocate for myself…and now off on leave due to the doctor being unwilling to listen.”* – Survey response from a female patient, 41–60 years old, who visited an urban ED

*“[Patient] was upset with this particular experience because he had to wait more than 12 hours in the ED, and he only saw an NP instead of a doctor. [Patient] said that normally, the NPs are great, but they weren’t able to give him the proper diagnosis, which led him to more pain long-term.”* – Survey response from a male patient, ≥61 years old, who visited an urban ED

6. Continuity of care

Patients emphasized the importance of receiving information on follow-up care. They valued regular check-ins and receiving what they considered an appropriate level of follow-up.

*“[Patient] was in for IV treatment…he was called at home to be checked in on and had a good level of care and follow-up…”* – Survey response from a male patient, ≥61 years old, who visited a rural ED

On the contrary, several patients reported not knowing who to contact for follow-up care, the timeline of when they would receive follow-up care information, or what the instructions were to manage their medical concerns outside of the ED

*“[Patient] was a little confused about the follow-up care instructions and left without knowing for sure who he needed to see/where he needed to go.”* – Survey response from a male patient, 21–40 years old, who visited an urban ED

Many patients reported being told to go to their family physicians for follow-up care. However, this proved difficult for those who do not have a family physician or do not have regular and easy access to their family physician.

*“[Patient] was quite lost in terms of follow-up care since he does not have a GP, which might be something worth noting for EDs that do not provide comprehensive follow-up care.”* – Survey response from a male patient, 41–60 years old, who visited an urban ED

*“[Patient] was also told to follow up with her family doctor, which she said was not helpful since the wait times to see her GP are [also] excessive.”* – Survey response from a female patient, ≥61 years old, who visited a rural ED

## Discussion

This study presented qualitative data about care experience from a large sample of ED patients in Eastern Canada. Overall, 831 patients participated in this study, with the majority being females (62%) and aged 61 years or older (41%). Open-ended responses were provided by at least 82% of survey participants and constitute the primary qualitative dataset for this analysis. In addition, a small subset of five survey respondents participated in semi-structured interviews to provide further depth and contextual understanding of patient experience in the ED.

Six key themes emerged from the integrated analysis: (1) Mutual respect and trust in providers, (2) Timeliness of care, (3) Communication, (4) Comfort and accommodations, (5) Information sharing and decision making, and (6) Continuity of care. These themes have been reflected in previous studies on patient experience [4,7,8] and patient-centred care [9,10].

### Integrating the theoretical framework

Several patients reported feeling dismissed or ‘not taken seriously’ by the providers, which led to feelings of disrespect and mistrust in the health system. In SI terms, patients interpret providers’ words, tone and body language as symbols of whether they are valued as people rather than cases, and how patients feel when care is delivered is important in shaping their experiences [1,26]. Some patients had a better experience when seen “quicker than normal,” while others waited for hours in overcrowded waiting rooms, feeling hungry, thirsty, and in pain, which created mistrust in the health system and a sense of hopelessness. Timeliness is a key context in which patients read the organization’s attitude toward them and adjust their expectations and trust accordingly [27]. Communication is closely aligned with the theme of mutual respect and trust; patients interpret not only information but also relational cues: empathy, attentiveness, eye contact, and openness to questions [27]. They feel appreciated when providers listen attentively but discouraged from seeking care when they are rushed or not given information in a way they understand.

Physical and environmental arrangements (privacy, noise, seating, accessibility) function as symbols of care or neglect [27]. Unhelpful providers and uncomfortable environments led to negative experiences, influencing whether patients feel safe to disclose sensitive information. Similarly, patients receiving adequate information and being able to participate in decisions about their care is a symbol of being in control and contributes to relational autonomy [28,29]. Finally, receiving follow up information helped patients not feel ‘lost’ and count on the health system for continuous quality care, thus increasing their reliability and trust in the system [30].

### Comparison to previous literature on patient experience

In our study, many patients reported feeling disrespected and having limited trust in providers because of how they interacted with them; adult female patients (aged >20 years) raised such concerns more often than male patients. Studies have shown that treating patients with respect and empathy improves their satisfaction, clinical safety, and effectiveness [4,7,8,31,32]. Acknowledging patients’ needs and exhibiting empathy and attentiveness have also been recognized as facilitators of patient-centred care [9,33]. Patients also expressed concerns over lengthy wait times, consistent with previous literature [7,8,34]. Timely care is one of the strongest determinants of patient satisfaction and care experience, increasing patient loyalty, trust in the health system, medication adherence, and overall clinical outcomes [7,8,34]. Overcrowding, resulting from a gap between demand for care and available resources, is one of the prominent causes of lengthy wait times [5,35,36]. Staff-patient communication is another strong determinant of patient experience [7–9]. Ineffective communication harms patients’ health and well-being and contributes to their aggressive behaviour toward providers [37,38].

Another construct of patient experience was comfort and accommodations. Patients found waiting rooms confined, with uncomfortable chairs and no availability of snacks or water nearby. Curtained cubicles affected privacy, and patients also reported a need for additional accommodations for those with mental health issues, substance issues, or behavioural problems. Studies have shown that older ED users (aged ≥65 years) often report discomfort due to hunger, thirst, limited privacy, and feeling cold from the ED environment and hospital gowns [39,40]. However, in our study, concerns about discomfort were raised by patients across all age groups. Finally, patients reported a negative experience due to insufficient follow-up care. One study showed that when male patients are contacted for a follow-up phone call, they perceive their ED visit more positively [41,42]. Such phone calls also allow providers to clarify instructions, coordinate referrals, and recognize patients needing additional support [41,42]. Studies have also shown that scheduling a follow-up appointment before leaving the ED ensures better follow-up care [42]. However, it is important to note that limited access to primary care and the lack of resources outside the ED may restrict the provision of adequate follow-up care. PRPs agreed that these findings reflect their ED experience. Therefore, this study indicates significant areas for improvement in ED patient care. As the only study in NL on patient experience, our findings will serve as an essential resource for the NL health system in enhancing ED care.

### Limitations

Applying SI as a theoretical lens enabled a nuanced understanding of how patients personally experienced emergency care [24]. This approach provided deeper insight into the emotional and relational aspects of care, enhancing the patient-centred relevance of our findings, particularly within rural healthcare contexts. Nevertheless, this approach has several limitations. The use of SI guided the analytic focus, potentially focusing on certain dimensions of patient experience, such as interpersonal dynamics, while underemphasizing others, such as systemic or structural factors [24]. Additionally, the multifaceted nature of healthcare experiences meant that some participant responses could align with multiple thematic categories. All themes were refined through iterative team discussions and consensus-building to ensure analytic clarity and rigor [18,23]. Finally, while no new themes emerged during the transition from open-ended survey responses to interview data, the conclusion that data saturation had been reached was ultimately a subjective judgment based on comprehensive tracking of theme recurrence across data sources, regular discussions, and ongoing consultation with PRPs.

Given the self-reported nature of the data, several potential sources of bias could have affected the validity and reliability of the findings. There is a possibility of social desirability bias, wherein participants may provide positive, socially desirable answers [43]. Our surveys and semi-structured interviews guaranteed anonymity and confidentiality to encourage honest responses. RAs who collected data were trained to conduct interviews ethically [18,23]. However, they paraphrased some patient responses, which could have introduced interviewer bias. Random sampling and high response rates were used to mitigate selection bias, ensuring a representative population sample [44]. The time gap between the survey and interviews may have led to recall bias, with patients potentially mixing details of their ED visit with similar experiences [18,23]. This paper presents a subset of findings from a larger qualitative analysis and does not capture the full spectrum of themes identified during the control period of the study. The team’s selective focus on data supporting the study hypothesis could have also resulted in confirmation bias. We addressed this by having five researchers of different ages, genders, backgrounds, and ethnicities and consulting PRPs to determine whether the research findings represent their lived experiences.

In terms of the ED setting, our results were similar across both urban and rural EDs. However, our prior quantitative studies have shown that patients had worse experience with privacy [18] and waiting times [23] in the urban EDs included in this study. Evidence also shows that age [40], gender [12] and cultural backgrounds [45] affect the patient experience. Particularly in Canada, there has been an increase in older patients awaiting long-term care placement in acute hospitals and EDs, often resulting in worsening conditions and a negative patient experience [46,47]. Although the majority of our participants were aged 61 years or older (41%), we did not identify any themes related to this issue or any differences in experience by age in general. Our results also did not show any differences among patients of different genders, except for female patients, raising more concerns about mutual respect and trust in providers. This finding should be interpreted with caution, as the sample included a higher proportion of female participants (62%) than males (32%), which may have influenced this finding and highlights the need for future research using more gender inclusive measures. In addition, the lack of differences related to patient characteristics may be due to the demographic context of NL. According to the 2021 Census, 23.6% of the total population of NL is aged 65 years or more, are mostly English (34.9%), Canadians (24.9%) or Irish (24.3%) and only a small population is gender diverse [16,48]. Finally, studies have shown differences in patient experiences between those admitted to hospital and those discharged from the ED [49]. However, our data did not show any noticeable differences in experience among patients who were admitted, discharged or transferred [49]. Therefore, future research needs to focus on these factors for a further in-depth analysis of patient experience.

### Practical recommendations

Discussions with PRPs resulted in identifying a need for regular training programs for providers focused on improving patient experience and the provision of PCC. However, such training should be viewed as complementary to addressing system-level challenges such as overcrowding, staffing shortages, and inadequate funding. We also recommend hiring patient navigators as patients’ companions during the ED visit. These navigators would assist with comfort needs, such as accessing water, snacks, or mobility aids, and facilitate access to private spaces for individuals requiring additional support, including those with mental health conditions or substance use concerns. Ensuring there is a water fountain and vending machine closer to the ED waiting rooms is also a practical and accessible initial step toward improving the patient experience. However, patients should be clearly informed if they need to refrain from eating or drinking, and the vending machines and water fountains should include visible signage advising patients to consult their care provider before consuming anything while waiting for care.

Due to the unpredictable nature of ED settings, regular communication with patients in the waiting room, including periodic reassessment of vital signs when necessary, and as per existing protocols, should be prioritized. Improving transparency around the triage process, alongside system-level efforts to address waiting room comfort, may help reduce patient anxiety and improve experience while awaiting care. Improving triage practices to better accommodate vulnerable populations, such as implementing a designated triage area for patients with disabilities to ensure added privacy, could also improve the overall ED experience. Finally, we recommend reviewing these findings with hospital patient and family advisory councils across the province. This process can guide decision-makers in identifying which improvements are immediately actionable, which require further planning, and which align with the priorities of local patients. Ultimately, such changes should be driven by patient priorities and validated through consultation with local advisory groups.

## Conclusion

Our findings highlight key areas of improving ED care, such as fostering mutual respect and trust between patients and providers, delivering timely care, improving communication, providing enhanced comfort and accommodations, including patients in decision-making, and giving adequate instructions on follow-up care. These improvements are crucial for creating a more supportive and efficient patient experience and providing patient-centred care.

## Supporting information

S1 File Consolidated criteria for reporting qualitative studies (COREQ): 32-item checklist. (DOCX)

S2 File SurgeCon Patient Emergency Department Experience and Satisfaction Survey. (DOCX)

S3 File SurgeCon Patient Emergency Department Experience & Satisfaction Interview Guide. (DOCX)
