# Supplementary material for: Voices from the emergency department: A theoretical framework analysis on patient experiences of care in emergency departments of Newfoundland and Labrador, Canada
Source: PLoS One. 2026 Feb 9;21(2):e0342555. doi: 10.1371/journal.pone.0342555 (PMC12885285; doi:10.1371/journal.pone.0342555)
Supplement: S1 File — (DOCX) [file pone.0342555.s001.docx]

**Voices from the Emergency Department: A Theoretical Framework Analysis on Patient Experiences of Care in Emergency Departments of Newfoundland and Labrador, Canada**

**Table:** Consolidated criteria for reporting qualitative studies (COREQ): 32-item checklist

| No. | Item | Guide questions/description | Response |
| --- | --- | --- | --- |
| 1. | Interviewer/facilitator | Which author/s conducted the interview or focus group? |  |
| 2. | Credentials | What were the researcher’s credentials? E.g. PhD, MD |  |
| 3. | Occupation | What was their occupation at the time of the study? |  |
| 4. | Gender | Was the researcher male or female? |  |
| 5. | Experience and training | What experience or training did the researcher have? |  |
| 6. | Relationship established | Was a relationship established prior to study commencement? | NA |
| 7. | Participant knowledge of the interviewer | What did the participants know about the researcher? e.g. personal goals, reasons for doing the research | NA |
| 8. | Interviewer characteristics | What characteristics were reported about the interviewer/facilitator? e.g. Bias, assumptions, reasons and interests in the research topic | NA |
| 9. | Methodological Orientation and Theory | What methodological orientation was stated to underpin the study? e.g. grounded theory,  discourse analysis, ethnography, phenomenology, content analysis |  |
| 10. | Sampling | How were participants selected? e.g. purposive, convenience, consecutive, snowball |  |
| 11. | Method of approach | How were participants approached? e.g. face-to-face, telephone, mail, email |  |
| 12. | Sample size | How many participants were in the study? |  |
| 13. | Non-participation | How many people refused to participate or dropped out? Reasons? |  |
| 14. | Setting of data collection | Where was the data collected? e.g. home, clinic, workplace |  |
| 15. | Presence of non-participants | Was anyone else present besides the participants and researchers? |  |
| 16. | Description of sample | What are the important characteristics of the sample? e.g. demographic data, date |  |
| 17. | Interview guide | Were questions, prompts, guides provided by the authors? Was it pilot tested? |  |
| 18. | Repeat interviews | Were repeat interviews carried out? If yes, how many? | NA |
| 19. | Audio/visual recording | Did the research use audio or visual recording to collect the data? |  |
| 20. | Field notes | Were field notes made during and/or after the interview or focus group? | NA |
| 21. | Duration | What was the duration of the interviews or focus group? |  |
| 22. | Data saturation | Was data saturation discussed? |  |
| 23. | Transcripts returned | Were transcripts returned to participants for comment and/or correction? | NA |
| 24. | Number of data coders | How many data coders coded the data? |  |
| 25. | Description of the coding tree | Did authors provide a description of the coding tree? | NA |
| 26. | Derivation of themes | Were themes identified in advance or derived from the data? |  |
| 27. | Software | What software, if applicable, was used to manage the data? |  |
| 28. | Participant checking | Did participants provide feedback on the findings? |  |
| 29. | Quotations presented | Were participant quotations presented to illustrate the themes / findings? Was each quotation identified? e.g. participant number |  |
| 30. | Data and findings consistent | Was there consistency between the data presented and the findings? |  |
| 31. | Clarity of major themes | Were major themes clearly presented in the findings? |  |
| 32. | Clarity of minor themes | Is there a description of diverse cases or discussion of minor themes? | NA |
