# Supplementary material for: Voices from the emergency department: A theoretical framework analysis on patient experiences of care in emergency departments of Newfoundland and Labrador, Canada
Source: PLoS One. 2026 Feb 9;21(2):e0342555. doi: 10.1371/journal.pone.0342555 (PMC12885285; doi:10.1371/journal.pone.0342555)
Supplement: S2 File — (DOCX) [file pone.0342555.s002.docx]

Patient Emergency Department Experience & Satisfaction Survey

Start of Block: Introduction

Q1.1 Patient Log Form Study ID

________________________________________________________________

Q1.2 This survey includes questions that were adapted from the *Ontario Emergency Department Patient Experience of Care Survey*, the *CIHI Canadian Patient Experiences Survey*, the *Press Ganey Emergency Department Survey*,  and the *NHS Accident and Emergency Department Questionnaire*.     **Note to Interviewer:**   **Avoid influencing answers by making comments or giving opinions.** **Be patient with the participants.** **Encourage participants to answer all questions.** **Remain as objective as possible. Ensure you have a patient log form ready.** **Read the following to the participant before commencing the survey.**

Q1.3
Hello, I am (say your name). I am an Eastern Health employee and part of a research team from Memorial University and Eastern Health who are developing an emergency department management system called ‘SurgeCon’.

The goal of the study is to reduce emergency department wait times, improve patient satisfaction and experiences, and improve the value of emergency healthcare services.

You have been invited to complete a telephone interview because you recently visited one of four Eastern Health emergency departments that are currently taking part in this study.

Taking part in this study is voluntary.  You may choose to take part, or you may choose not to take part in this study.  You also may change your mind at any time. Whatever you choose it will not affect your usual care and you will not lose any benefits to which you are entitled.

There is an optional prize draw for patients deciding to participate in the study. Prizes to be won are iPads or gift cards.

Q1.4 Should you agree to participate in this interview, all data collected from this survey will be anonymized and no identifiable information will be stored alongside it. Your identity will be kept anonymous to all research staff except for the interviewer conducting the interview. The information you provide in this interview is important. A research team from Memorial University will analyze your responses to the questions included in the survey. The results of this analysis will provide the research team and Eastern Health valuable information related to the perspectives of patients on different aspects of emergency care. This information will be used to improve emergency care services so that they address the needs and priorities of patients.  The results of this study will be presented to decision makers in our province, published in peer-reviewed academic journals, and presented at scientific meetings. When the results of this study are published in journals or presented at scientific meetings, your name and other personal information will not be used in any publications or presentations.

Q1.5 During the interview, you may feel uncomfortable or experience some anxiety, emotional and/or psychological distress due to the nature of the questions. You can skip questions, take a break or stop answering at any time. We can provide contact information to resources that provide psychological support. **Note to interviewer: Provide the contact numbers if the participant requests them.**   24 hour Province-Wide Mental Health Crisis Phone Line: Tel: (709) 737-4668     Toll-free: 1-888-737-466   You can contact the office of the health research ethics authority, lead researcher, or project manager if you have any questions related to the study, your participation in the study, your rights as a participant or any other questions or concerns. **Note to interviewer: Provide the contact numbers if the participant requests them.**

Q1.6 Do you consent to participate in this study, given the information I just provided? Note to interviewer: Record the response to this question on the patient log form.

Q1.7 Would you like to include your name in the annual prize draw? You will remain anonymous during the prize draw. Note to interviewer: Record the response to this question on the patient log form.

- Yes
- No

Q1.8 We can send you a digital copy of the informed consent document via email so you can carefully review your rights as a participant, the risks and benefits of participating in the study and additional information related to the study itself and those you can contact should you have any additional questions.

Would you like us to send you the informed consent document?

- Yes
- No

Q1.9 Can you please provide your email address? **Note to interviewer: Please record response in patient log form and make sure you read the email address back to the patient to ensure it was correctly recorded.**

Q1.10 **Note to Interviewer: Please record the following information on the patient log form 1. Date/Time**
**2. Name of the participant**
**3. Name of the legal guardian (if applicable)**
**4. Patient Age Group**
**5. Patient Gender**
**6. Record the participant's informed consent response**
**7. Record the participant's response for the prize draw.**
**8. Participant Email (Required for Consent Form/Prize Draw Email)**
**9. Name of the ER they visited (**Cross reference the ER name with the name of the facility used to select the participant**)**
**10. Date/Time of ED Visit**(**Cross reference the Date/Time of day used to select the participant**) **11. Please record that the Consent Form was read and explained to the participant before receiving the participant’s consent, and the participant has knowledge of the research project and appeared to understand it.**
 
**When you have completed the interview - please archive the patient log form for record keeping purposes.**

End of Block: Introduction

Start of Block: ED Visit Confirmation

Q2.1 Can you confirm which hospital you visited for your emergency? **Note to Interviewer: This information should be consistent with the information displayed on Meditech. If different, please ensure the patient is aware of the specific ED visit you are calling about.**

▼

Q2.2 Can you confirm the date and time of your emergency department visit? Note to Interviewer: This information should be consistent with the information displayed on Meditech. If different, please ensure the patient is aware of the specific ED visit you are calling about.

________________________________________________________________

End of Block: ED Visit Confirmation

Start of Block: Coordination of Care - Emergency Department Arrival

Q3.1 Thinking about this visit, what was the main reason why you went to the emergency department?

- An accident or injury
- A new health problem
- An ongoing health condition or concern
- The emergency department was the only healthcare service available at the time of my visit
- Other (Please Specify) __________________________________________________

Q3.2 Were you assessed by the triage nurse?

- Yes
- No
- Not Sure

Q3.3 Did you leave the emergency department before being seen by a doctor or nurse practitioner?

- Yes
- No

Q3.4 Do you live with a disability? This could include hearing, vision, mobility, flexibility, dexterity, pain, learning, mental health, memory and developmental disabilities.

- Yes
- No
- I would prefer not to say

Q3.5 Were you temporarily disabled or impaired as a result of your medical emergency?

- Yes
- No
- I would prefer not to say

Q3.6 Does your disability/temporary impairment require(d) the assistance of a support person when visiting the emergency department?

- Yes
- No

Q3.7 Were you alone during your emergency department visit?

- Yes
- No

Q3.8 Did any of the emergency department staff ask if you required a support person or whether you needed accommodations to overcome specific barriers to accessibility or receiving care?

- Yes
- No

Q3.9 Can you confirm which accommodations were provided during your visit?

- Wheel chair
- Support person/porter
- Buzzer / Assistance with using washroom, dressing/undressing, eating/drinking
- I would prefer not to say
- I did not require any accommodations
- Other __________________________________________________
- N/A (Didn't Ask / Wasn't Offered)

Q3.10 Was the wheel chair provided for your emergency visit comfortable?

- Yes
- No
- Can't Remember

Q3.11 Did the discomfort from wheel chair aggravate your medical emergency?

- Yes
- No
- Can't Remember

Q3.12 Thinking about this visit, what was the main reason why you went to the emergency department?

- An accident or injury
- A new health problem
- An ongoing health condition or concern
- The emergency department was the only healthcare service available at the time of my visit
- Other (Please Specify) __________________________________________________

Q3.13 Can you confirm which accommodations were provided during your visit?

- Wheel chair
- Support person/porter
- Buzzer / Assistance with using washroom, dressing/undressing, eating/drinking
- I would prefer not to say
- I did not require any accommodations
- Other __________________________________________________
- N/A (Didn't Ask / Wasn't Offered)

End of Block: Coordination of Care - Emergency Department Arrival

Start of Block: Coordination of Care - During the Emergency Visit

Q4.1 From the time you first arrived at the Emergency Department, how long did you wait before being examined by a provider (doctor or nurse practitioner)? **Interviewer Note: For the remainder of this interview 'doctor' will be used to refer to physicians, nurse practitioners and/or their delegate (e.g. Resident)**

- I did not have to wait
- 1 - 30 minutes
- 31 - 60 minutes
- More than 1 hour but less than 2 hours
- More than 2 hours but less than 4 hours
- More than 4 hours
- Don't Know / Can't Remember

Q4.2 From the time you first arrived at the Emergency Department, how long did you wait before leaving? **Interviewer Note: For the remainder of this interview 'doctor' will be used to refer to physicians, nurse practitioners and/or their delegate (e.g. Resident)**

- 1 - 30 minutes
- 31 - 60 minutes
- More than 1 hour but less than 2 hours
- More than 2 hours but less than 4 hours
- More than 4 hours
- Don't Know / Can't Remember

Q4.3 Were you told how long you would have to wait to be examined by a provider (doctor or nurse practitioner)?

- Yes, but the wait was shorter
- Yes, and I had to wait about as long as I was told
- Yes, but the wait was longer
- No, I was not told
- Don't know / Can't remember
- N/A

Q4.4 Overall, what was the total length of your visit to the ED?

▼ 15 minutes or less ... 8+ hours

Q4.5 Using any number from 0 to 10, where 0 is extremely dissatisfied and 10 is extremely satisfied, how satisfied were you with the amount of time before being seen by a doctor or nurse practitioner?

|  | Extremely dissatisfied | Somewhat dissatisfied | Neither satisfied nor dissatisfied | Somewhat satisfied | Extremely satisfied |
| --- | --- | --- | --- | --- | --- |

|  | 0 | 1 | 2 | 3 | 4 | 5 | 6 | 7 | 8 | 9 | 10 |
| --- | --- | --- | --- | --- | --- | --- | --- | --- | --- | --- | --- |

| Scale | 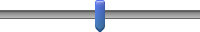 |
| --- | --- |

Q4.6 Using any number from 0 to 10, where 0 is extremely dissatisfied and 10 is extremely satisfied, how satisfied were you with total time spent in the emergency department?

|  | Extremely dissatisfied | Somewhat dissatisfied | Neither satisfied nor dissatisfied | Somewhat satisfied | Extremely satisfied |
| --- | --- | --- | --- | --- | --- |

|  | 0 | 1 | 2 | 3 | 4 | 5 | 6 | 7 | 8 | 9 | 10 |
| --- | --- | --- | --- | --- | --- | --- | --- | --- | --- | --- | --- |

| Scale | 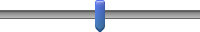 |
| --- | --- |

Q4.7 While waiting for care, did the emergency department staff check to see how you were doing?

- Yes
- No
- Don't Know / Can't Remember

End of Block: Coordination of Care - During the Emergency Visit

Start of Block: Coordination of Care - Leaving the Emergency Department

Q5.1 Were you admitted to the hospital as a result of this emergency department visit?

- Yes
- No
- N/A

Q5.2 Using any number from 0 to 10, where 0 is extremely dissatisfied and 10 is extremely satisfied, how satisfied were you with the wait time before being transferred to an inpatient unit?

|  | Extremely dissatisfied | Somewhat dissatisfied | Neither satisfied nor dissatisfied | Somewhat satisfied | Extremely satisfied |
| --- | --- | --- | --- | --- | --- |

|  | 0 | 1 | 2 | 3 | 4 | 5 | 6 | 7 | 8 | 9 | 10 |
| --- | --- | --- | --- | --- | --- | --- | --- | --- | --- | --- | --- |

| Scale | 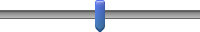 |
| --- | --- |

End of Block: Coordination of Care - Leaving the Emergency Department

Start of Block: Overall Impression - During the Emergency Visit

Q6.1 Using any number from 0 to 10, where 0 is the worst possible experience and 10 is the best possible experience, what number would you use to rate your experience receiving care during this emergency department visit?

|  | Worst experience possible | Best experience possible | Not Applicable |
| --- | --- | --- | --- |

|  | 0 | 1 | 2 | 3 | 4 | 5 | 6 | 7 | 8 | 9 | 10 |
| --- | --- | --- | --- | --- | --- | --- | --- | --- | --- | --- | --- |

| Scale | 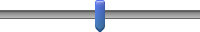 |
| --- | --- |

Q6.2 What would you change in the care you received, if at all?

________________________________________________________________

________________________________________________________________

________________________________________________________________

________________________________________________________________

________________________________________________________________

End of Block: Overall Impression - During the Emergency Visit

Start of Block: Overall Impression - Leaving the Emergency Department

Q7.1 Overall, did you get the care you expected, why or why not?

- Yes
- No (If not, why?) __________________________________________________

End of Block: Overall Impression - Leaving the Emergency Department

Start of Block: Cost to Patients - Emergency Department Arrival

Q8.1 Were you taken to the hospital in an ambulance?

- Yes
- No

Q8.2 How far away were you from the hospital?

- 0 - 30 minutes
- 31 - 59 minutes
- 1 - 2 hours
- > 2 hours
- > 3 hours
- > 4 hours
- I was driven to the ED in an ambulance

Q8.3 How long did it take for the ambulance to arrive

- 0-30 minutes
- 31-59 minutes
- 1-2 hours
- >2 hours
- >3 hours
- >4 hours

Q8.4 Were there other emergency departments closer than the one you visited?

- Yes
- No
- Don't Know / I was driven to the ED in an ambulance

Q8.5 Why did you decide to travel to an emergency department that was further away?

________________________________________________________________

End of Block: Cost to Patients - Emergency Department Arrival

Start of Block: Cost to Patients - During the Emergency Visit

Q9.1 Did you or a family member/friend lose any income as a result of your latest emergency department visit outside of gas and parking? **(Note to interviewer: Select all that apply, and request specific amount for each applicable category.)**

- Missed hours of work (If yes, how many hours?) __________________________________________________
- Hotel (If yes, how much?) __________________________________________________
- Babysitter/Caretaker (If yes, how much?) __________________________________________________
- Taxi (If Yes, how much?) __________________________________________________
- Other Financial Problems (Please specify) __________________________________________________
- No (If none apply - select this option)
- I would prefer not to say

End of Block: Cost to Patients - During the Emergency Visit

Start of Block: Cost to Patients - Leaving the Emergency Department

Q10.1 Overall, how much did the emergency department visit cost you? (This would include food, hotel, transportation, babysitters/caretakers, gas, parking, missed hours of work, etc.)

- $1 - $199
- $200 - $499
- $500 - $999
- $1,000 - $5,000
- More than $5,000
- I cant remember
- There was no cost associated with this emergency visit

End of Block: Cost to Patients - Leaving the Emergency Department

Start of Block: Amenities & Physical Comfort - During the Emergency Visit

Q11.1 Was the waiting room large enough for everyone to sit comfortably (while also socially distancing)? **(Note to interviewer: Only refer to social distancing while COVID-19 pandemic regulations are still in effect.)**

- Yes
- No
- Can't Remember

Q11.2 Did you have any concerns related to COVID-19 (e.g. frequency of cleaning, cleanliness of waiting room/bathroom, etc.)?

________________________________________________________________

________________________________________________________________

________________________________________________________________

________________________________________________________________

________________________________________________________________

Q11.3 Using any number from 0 to 10 where 0 is extremely dissatisfied and 10 is extremely satisfied, how satisfied were you with the emergency department’s waiting room environment?

|  | Extremely dissatisfied | Somewhat dissatisfied | Neither satisfied nor dissatisfied | Somewhat satisfied | Extremely satisfied |
| --- | --- | --- | --- | --- | --- |

|  | 0 | 1 | 2 | 3 | 4 | 5 | 6 | 7 | 8 | 9 | 10 |
| --- | --- | --- | --- | --- | --- | --- | --- | --- | --- | --- | --- |

| Scale | 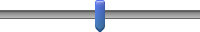 |
| --- | --- |

Q11.4 Did you feel the emergency department was overcrowded during your visit?

- Yes
- No
- Can't Remember

Q11.5 Do you have any suggestions on how to improve the emergency department's environment?

________________________________________________________________

________________________________________________________________

________________________________________________________________

________________________________________________________________

________________________________________________________________

Q11.6 Did the emergency department doctors, nurses, other support staff show any concern for your comfort?

- Yes
- Somewhat
- No

Q11.7 Do you think the emergency department doctors, nurses, and other support staff did everything they could to help manage your health concern?

- Yes
- Somewhat
- No
- Can't say / Don't Know

Q11.8 Using any number from 0 to 10 where 0 is very slow and 10 is very fast, how quickly did staff respond to your requests for assistance? (e.g. glass of water, help to use washroom, pain medication, etc.)

|  | Very slow | Somewhat slow | Average | Somewhat fast | Very fast | Not Applicable |
| --- | --- | --- | --- | --- | --- | --- |

|  | 0 | 1 | 2 | 3 | 4 | 5 | 6 | 7 | 8 | 9 | 10 |
| --- | --- | --- | --- | --- | --- | --- | --- | --- | --- | --- | --- |

| Scale | 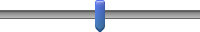 |
| --- | --- |

Q11.9 Were you given enough privacy when talking to staff, or when being examined or treated?

- Yes
- Somewhat
- No
- N/A

End of Block: Amenities & Physical Comfort - During the Emergency Visit

Start of Block: Emotional Support - During the Emergency Visit

Q12.1 Did you get the support you needed to help you with any anxieties, fears or worries during your visit? **(Note to interviewer: Please read the options for the patient to select)**

- Yes
- Somewhat
- No
- I did not experience any emotional distress
- Can't remember
- I would prefer not to say

End of Block: Emotional Support - During the Emergency Visit

Start of Block: Emotional Support - Leaving the Emergency Department

Q13.1 Did the Doctor or Nurse Practitioner tell you who to contact if you were worried about your health problem after leaving the ED?

- Yes
- No
- N/A, my health problem was solved
- N/A, left without being seen by Doctor or Nurse Practitioner
- I was told to return to the Emergency Department for Follow-Up Care
- I can't remember

End of Block: Emotional Support - Leaving the Emergency Department

Start of Block: Respect for Patient Preferences - During the Emergency Visit

Q14.1 During this emergency department visit, how often did nurses treat you with courtesy and respect?

- Always
- Usually
- Sometimes
- Never
- N/A

Q14.2 During this emergency department visit, did nurses spend enough time with you?

- Yes
- Somewhat
- No
- N/A

Q14.3 During this emergency department visit, how often did reception, custodial, administrative staff & paramedics treat you with courtesy and respect?

- Always
- Usually
- Sometimes
- Never
- N/A

Q14.4 Can you confirm if the following types of healthcare providers were involved in your care? **Note to Interviewer: Select all that apply.**

- Doctor
- Nurse Practitioner
- Resident
- I can't remember
- N/A

Q14.5 During this emergency department visit, how often did doctors treat you with courtesy and respect?

- Always
- Usually
- Sometimes
- Never
- N/A

Q14.6 During this emergency department visit, how often did the doctor(s) listen carefully to you?

- Always
- Usually
- Sometimes
- Never
- N/A

Q14.7 Were you given enough time to discuss your health or medical problem with the doctor?

- Yes
- Somewhat
- No
- N/A

Q14.8 During this emergency department visit, how often did the nurse practitioner treat you with courtesy and respect?

- Always
- Usually
- Sometimes
- Never
- N/A

Q14.9 During this emergency department visit, how often did the nurse practitioner listen carefully to you?

- Always
- Usually
- Sometimes
- Never
- N/A

Q14.10 Were you given enough time to discuss your health or medical problem with the nurse practitioner?

- Yes
- Somewhat
- No
- N/A

Q14.11 Did the members of your care team involve you in the plan for your care and treatment?

- Yes
- Somewhat
- No
- I was not well enough to be involved in decisions about my care
- N/A

Q14.12 Do you feel the care provider provided enough information related to the care and treatment plan chosen? **(Note to interviewer: Care and treatment may include lab tests, diagnostic imaging, diagnosis, medication, etc.)**

- N/A
- Yes
- No __________________________________________________

End of Block: Respect for Patient Preferences - During the Emergency Visit

Start of Block: Information & Education - During the Emergency Visit

Q15.1 During this emergency department visit, how often did staff explain things in a way you could understand?

- Always
- Usually
- Sometimes
- Never

Q15.2 When discussing your treatment plan, diagnosis, or plan for follow-up care, did emergency department staff ask you to confirm whether you fully understood?

- Yes
- No
- I can't remember
- N/A

Q15.3 Using any number from 0 to 10 where 0 represents not at all well and 10 represents extremely well, how well were you kept informed about delays impacting your care?

|  | Not well at all | Slightly well | Moderately well | Very well | Extremely well | Not Applicable |
| --- | --- | --- | --- | --- | --- | --- |

|  | 0 | 1 | 2 | 3 | 4 | 5 | 6 | 7 | 8 | 9 | 10 |
| --- | --- | --- | --- | --- | --- | --- | --- | --- | --- | --- | --- |

| Scale | 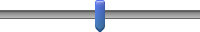 |
| --- | --- |

End of Block: Information & Education - During the Emergency Visit

Start of Block: Continuity & Transition - Leaving the Emergency Department

Q16.1 Before you left the emergency department, did someone discuss with you whether you needed follow-up care?

- Yes
- No

Q16.2 Did you feel you were provided with enough information to safely return home?

- Yes
- No (If not, can you please elaborate) __________________________________________________

End of Block: Continuity & Transition - Leaving the Emergency Department

Start of Block: Demography

Q17.1 Can you confirm your age? **(Note to interviewer: Please select applicable age group)**

- 0-5
- 6-10
- 11-15
- 16-20
- 21-25
- 26-30
- 31-35
- 36-40
- 41-45
- 46-50
- 51-55
- 56-60
- 61-65
- 66-70
- 71-75
- 76-80
- 81-85
- 86-90
- 91-95
- 96-100
- >100

Q17.2 Can you confirm your gender? **(Note to interviewer: Do not read options to patient)**

- Male
- Female
- Other (please state) __________________________________________________

End of Block: Demography

Start of Block: Conclusion

Q18.1 Is anything else you would like to tell us about your experience at the emergency department?    **Interviewer Note: Suggested questions:** **Was there anything particularly good about your visit to the Emergency Department? Was there anything that could have been improved? Any other comments?**

________________________________________________________________

________________________________________________________________

________________________________________________________________

________________________________________________________________

________________________________________________________________

Q18.2 Members of the Memorial University research team will be conducting a second in-depth interview with a subset of patients who have completed this telephone survey. The second interview will be approximately 45 minutes in length and include questions related but not limited to emotional support, healthcare provider trust and communication, financial barriers, and the department's environment and level of comfort. If you complete the interview you will receive a $25 Amazon gift card via email. Do you consent to being contacted by members of the Memorial University research team to complete a second interview?

- Yes
- No
- N/A

Q18.3 Interviewer Observation Notes: **(Note to interviewer: Please record any unexpected responses/events (e.g. interview interrupted) in this section.**

________________________________________________________________

________________________________________________________________

________________________________________________________________

________________________________________________________________

________________________________________________________________

Q18.4 **THANK YOU VERY MUCH FOR YOUR HELP**

End of Block: Conclusion
