# Supplementary material for: Voices from the emergency department: A theoretical framework analysis on patient experiences of care in emergency departments of Newfoundland and Labrador, Canada
Source: PLoS One. 2026 Feb 9;21(2):e0342555. doi: 10.1371/journal.pone.0342555 (PMC12885285; doi:10.1371/journal.pone.0342555)
Supplement: S3 File — (DOCX) [file pone.0342555.s003.docx]

**Patient Emergency Department Experience & Satisfaction Interview Guide**

Hello, I am (say your name). I am a Memorial University of Newfoundland staff member and part of a research team from Memorial University and Newfoundland and Labrador Health Services who are developing an emergency department management system called ‘SurgeCon’. The goal of the study is to reduce emergency department wait times, improve patient satisfaction and experiences, and improve emergency healthcare services. Do you have any questions before we begin? Just a reminder I’d like to record the interview; may I start the recording now? [Wait for response; if yes, continue] OK- I’m recording now.

Taking part in this study is voluntary.  You may choose to take part or you may choose not to take part in this study.  You also may change your mind at any time. Whatever you choose it will not affect your usual care and you will not lose any benefits to which you are entitled.
Should you agree to participate in this interview, all data collected from this survey will be anonymized and no identifiable information will be stored alongside it. Your identity will be kept anonymous to all research staff except for the interviewer conducting the interview.

The information you provide in this interview is important. A research team from Memorial University will analyze your responses to the questions included in the survey. The results of this analysis will provide the research team and Eastern Health valuable information related to the perspectives of patients on different aspects of emergency care. This information will be used to improve emergency care services so that they address the needs and priorities of patients.

During the interview, you may feel uncomfortable or experience some anxiety, emotional and/or psychological distress due to the nature of the questions. You can skip questions, take a break or stop answering at any time. We can provide contact information to resources that provide psychological support. **Note to interviewer: Provide the contact numbers if the participant requests them.** 24-hour Province-Wide Mental Health Crisis Phone Line:
 Tel: (709) 737-4668     Toll-free: 1-888-737-4668   You can contact the office of the health research ethics authority, lead researcher, or project manager if you have any questions related to the study, your participation in the study, your rights as a participant or any other questions or concerns. **Note to interviewer: Provide the contact numbers if the participant requests them.**
 

Do you consent to participate in this study, given the information I just provided? **Note to interviewer:** Record the response to this question on the patient log form.

Email address for $25 Amazon gift card:

**Opening Questions**

1-Can you confirm which hospital you visited for your emergency?

2-Can you confirm the date and time of your emergency department visit?

3-Why did you decide to come to the ED? Did you call your family physician before visiting the ED? (**NOTE:** If the patients answer “no”) Why not? (**NOTE:** If the patient answers “yes”) What happened when you called?

4- Do you prefer healthcare or treatment at the ED in comparison to your family physician **(NOTE:** If yes**)** Why? **(NOTE:** If no**)** Why not?

5-When you first arrived at the emergency department, how important was it for you to get care right away?

6- Age:

7- Gender:

8- Physician’s gender:

**Note to interviewer: Ask participants to explain their experiences in other EDs as well.**

**Respect and Trust/Emotional Support**

8- How important is the level of compassion doctors and nurses display while providing care? Is it more important than department efficiency? Explain your answer. (Follow-Up with Probing Questions)

Did the nursing staff appropriately address all your clinical needs? How did they deal with your situation/the situation of your loved one? Were doctors, nurses and other ED team members effective at resolving any issues encountered during your ED visit?

How satisfied were you with the way you were treated by emergency department staff? If you needed help, did you receive it as quickly as you wanted?

Can you tell me about your different encounters with physicians or nurses? How helpful were they in resolving your emergency? Were you satisfied with the outcome of the encounter/assessment? Can you provide an example of a situation where you were not satisfied with the result of the encounter?

How proactive were they in providing care and helping you move through the emergency care process?

Have you ever experienced an emergency department visit that went well and you felt well cared for?

**Communication**

9-Did you feel that ED staff listened to you and took into consideration your needs and priorities? Can you give an example? (Follow-Up with Probing Question)

1. What did you do to cope with any concerns or worries while you were in the ED? Did you feel you were able to talk to staff about your concerns? How did they respond to your concerns?

**Involvement of Patient/Family in Information Sharing and Decision Making**

10-How did you feel about the information ED staff provided you with with regard to your treatment plan or diagnosis? Did they provide information related to therapies or treatments? Did you feel involved in the decisions about your health? (Follow-Up with Probing Question)

1. Did you feel that you understood what was happening while in hospital? Have you ever experienced a situation where you felt you did not understand? Did staff help you understand your care plan or test/assessment results?

**Technical Determinants (i.e., Competence care needs)**

12-Can you provide your opinion on how well the emergency department was operating and their technical competence, and skills? Would you say emergency department staff seemed to be organized and following a pre-determined plan? How did you feel during clinical assessments and procedures? (Follow-Up with Probing Question)

1. How organized was your transfer from the emergency department into a hospital bed/inpatient unit?

2. Were emergency department staff effective in terms of relieving and managing your pain during clinical assessments and procedures?

**Financial Barriers**

13-How much money did you or a family member/friend have to spend on transportation/travel to get to the emergency department (taxi, hotel, parking, gas, etc.)? (Follow-Up with Probing Question)

1. Did you or a family member/friend have to pay a babysitter or caretaker to care for a family member as a result of your latest emergency department visit?
2. After being discharged from the emergency department, were there any additional expenses associated with getting back home outside of regular transportation?

**Comfort of Environment**

11-Would you please describe the waiting room? (e.g., lack of comfortable chairs, lack of privacy, noise, challenging wayfinding, the Internet, lots of clutter on walls). (Follow-Up with Probing Questions)

1. How did the waiting room environment impact your overall wellbeing or emotional wellbeing?

2.Was the waiting room large enough for everyone to sit comfortably?

3.How comfortable was the waiting room environment in which you were waiting for provider care (e.g. assessment room)?

4.What can be done to make the environment more suitable to patients and their families?

5. Did staff provide accurate and timely information regarding your wait time in the ED? If not, explain how you dealt with the uncertainty?

6. How would you describe the flow of patients through the emergency department?

7. Did you have an easy time finding your way around the ED? Could you please describe a circumstance in which you had a difficult time finding your way?

8- Could please describe a situation that your privacy was supported/not supported by the emergency department physical environment / layout?

9- Other factors we might want to consider:

- parking
- entry zone/arrival
- lighting
- temperature
- noise levels
- Internet
- furniture
- food services
- visual distractions
- colors in ED
- view from windows/existence of windows
- care zones, specialized rooms, etc

**Conclusion:**

14-What are your greatest ongoing difficulties in receiving emergency care?

15-Is there anything you would change with respect to the care you received?
